# Impact of diagnosis and treatment on response to COVID-19 vaccine in patients with *BCR-ABL1*-negative myeloproliferative neoplasms. A single-center experience

**DOI:** 10.1038/s41408-021-00579-0

**Published:** 2021-11-26

**Authors:** Daniele Cattaneo, Cristina Bucelli, Francesca Cavallaro, Dario Consonni, Alessandra Iurlo

**Affiliations:** 1 Hematology Division, Foundation IRCCS Ca’ Granda Ospedale Maggiore Policlinico, Milan, Italy; 2 Department of Oncology and Hemato-Oncology, University of Milan, Milan, Italy; 3 Epidemiology Unit, Foundation IRCCS Ca’ Granda Ospedale Maggiore Policlinico, Milan, Italy

**Keywords:** Myeloproliferative disease, Preventive medicine

Dear Editor,

The SARS-CoV-2 coronavirus infection causing the coronavirus disease 2019 (COVID-19) is a highly contagious and life-threatening disease critically associated with a high rate of respiratory failure, thrombo-hemorrhagic complications, and death, mainly due to an abnormal inflammatory response [1].

As *BCR-ABL1*-negative myeloproliferative neoplasms (MPN) patients are prone to both thrombosis and bleeding, the occurrence of COVID-19 calls for special care. With the aim to assess the prevalence of COVID-19 in *BCR-ABL1*-negative MPN subjects, the GIMEMA (Gruppo Italiano Malattie Ematologiche dell’Adulto) group conducted a survey of 34 Italian centers. Out of a cohort of 13.248 cases, a total of 1.095 patients were treated with ruxolitinib, 75.7% for myelofibrosis (MF) and 24.3% for polycythemia vera (PV). Thirty-six subjects (33.6% of patients tested) were found positive for COVID-19: 13 (36%) were asymptomatic, 13 (36%) had flu-like symptoms and ten (27.8%) were affected by COVID-19-related pneumonia. Eight COVID-19 positive patients died with a death rate of 22%. According to this survey, the incidence of COVID-19 infection in *BCR-ABL1*-negative MPNs appears to be rather low and a possible protective function of ruxolitinib could not be ruled out [2].

A subsequent study by the European LeukemiaNet collected 175 MPN patients with COVID-19 during the first wave of the pandemic, from February to May 2020, in 38 international hematologic centers [3]. Among the MPN phenotypes, patients with MF were the majority (44.6%) in comparison with ET (29.1%) and PV (26.3%); moreover, MF was characterized by a higher mortality (48%) *vs.* both ET (25%) and PV (19%). When compared with the general COVID-19 population, the mortality ratio in this study was at least two to three times higher than the one reported by the Johns Hopkins University in the same period and similar to that recorded in other hematologic malignancies [4–6]. With regards to therapy ongoing at COVID-19 diagnosis, while multivariable and propensity score matching analyses found an increased risk of death in patients who abruptly discontinued ruxolitinib treatment, this effect was not reported in patients treated with hydroxycarbamide (HU). The development of a severe inflammatory reaction consequent to sudden JAK inhibitors’ suspension could possibly explain these findings [3].

About the issue of response to COVID-19 vaccine in patients with hematological malignancies, several studies have already demonstrated a substantially reduced seroconversion rate, particularly in heavily treated groups and in those with aggressive disease, marked cytopenias, or B-cell neoplasms [7–9].

On the contrary, patients with chronic myeloid neoplasms, including MPN and chronic myeloid leukemia (CML), seemed to show higher seroconversion rates than those reported in the former groups: more in details, reasonably high seroconversion rates following a single vaccine dose were observed in patients with CML and in MPN patients receiving interferon. Instead, not surprisingly owing to its immunomodulatory properties, in MPN patients treated with ruxolitinib an impaired early response to SARS‐CoV‐2 vaccine was found as compared to healthy adults of a similar age group [10–13].

In this study, we prospectively evaluated at a single center in Milan, Italy 62 MPN patients undergoing SARS-CoV-2 mRNA vaccination from March until June 2021 selected and prioritized for vaccination as per indications of the Italian Ministry of Health [https://www.gazzettaufficiale.it/eli/gu/2021/03/24/72/sg/pdf]. The vaccines used were the Moderna and Pfizer-BioNTech vaccine in 40 (64.5%) and 22 (35.5%) cases, respectively.

Patients were sampled and tested for anti-Spike and anti-Nucleocapside IgG titer after a median time from the second vaccine dose of 5.3 weeks. A cut-off value of test positivity was established for each antibody type according to manufacturer’s instructions; patients above the upper cut-off level were considered as “responders”, and those below as “non responders”.

We used chi-squared test to compare proportions of responders (seroconversion) across variable categories. Then, we evaluated the relative risk (RR) and 95% confidence interval (CI) of seroconversion with a multivariable robust regression model with robust standard error [14]. Among responders, we compared anti-Spike levels across variable categories with Mann-Whitney test. Finally, we evaluated log(anti-Spike titer) with a multiple linear regression model. In both cases the models contained the following variables: vaccine type, gender, age at vaccination (<70, ≥70 years), body mass index (BMI) (<25, ≥25), MF diagnosis (no, yes), ruxolitinib (no, yes), and time from start of ongoing therapy to vaccination (<3, ≥3 years).

Median age at vaccination was 71.9 years, 35 (56.5%) were male, and median BMI was 24.5. Their main clinical features are reported in Table 1.

**Table 1:** Patients’ characteristics.

Variables	Patients (*N*. 62)
Age at vaccination (years), median (range)	71.9 (33.7–86.3)
Male, *n* (%)	35 (56.5)
BMI (kg/m^2^), median (range)	24.5 (19.1–37.4)
*Diagnosis, n (%)*	
PV	21 (33.9)
ET	11 (17.7)
Myelofibrosis	26 (41.9)
PMF	15 (24.2)
SMF	11 (17.7)
MPN, U	4 (6.5)
*Driver mutations, n (%)*	
*JAK2*V617F	49 (79.1)
*CALR*	10 (16.1)
*MPL*	2 (3.2)
Triple negative	1 (1.6)
*Cytoreductive therapy at vaccination, n (%)*	
Hydroxyurea	15 (24.2)
Anagrelide	3 (4.8)
Interferon	4 (6.5)
Givinostat	3 (4.8)
Ruxolitinib	30 (48.4)
Other JAK inhibitors	2 (3.2)
No therapy	5 (8.1)
Ruxolitinib daily dose (mg), median (range)	20 (10–40)
*Vaccine type, n (%)*	
BNT162b2 (Pfizer-BioNTech)	22 (35.5)
mRNA-1273 (Moderna)	40 (64.5)
Time from MPN diagnosis to vaccination (years), median (range)	9.7 (0.1–36.2)
Time from ongoing treatment start to vaccination (years), median (range)	3.1 (0.1–18.5)

*BMI* body mass index, *PV* polycythemia vera, *ET* essential thrombocythemia, *PMF* primary myelofibrosis, *SMF* secondary myelofibrosis, *MPN, U* myeloproliferative neoplasm, unclassifiable.

In particular, there were 21, 11 and 26 patients with PV, ET and MF (either primary—15 patients, or secondary MF—11 patients). All cases except five were on active treatment, with a median number of lines of therapy of 1 (range: 1–3): they included 30 patients on ruxolitinib, 15 on HU, 4 on interferon alpha, and 3 on anagrelide, with a median time from MPN diagnosis to vaccination and from beginning of ongoing therapy to vaccination of 9.7 and 3.1 years, respectively.

Specifically focusing on ruxolitinib, its current median dose was 20 mg daily (range: 10–40 mg), with a median duration of 3.0 years (range: 0.1–7.2).

Out of 62 subjects, 14 (22.6%) had non-detectable anti-Spike (i.e., less than the limit of quantification of our laboratory of 0.4 U/mL). Four (6.5%) patients out of 62 had positive anti-nucleocapside antibodies, three of them reported a previous SARS-CoV-2 infection. Two of them had MF, three were vaccinated with Pfizer-BioNTech and one with Moderna. All seroconverted for anti-Spike with a high titer (i.e., >7500 U/mL or >12500 U/mL, old and new upper laboratory limits).

There was no difference between the two vaccine types in responders rate (*p* = 0.21) nor in antibody titers (*p* = 0.88) (Table 2, *third column*). The likelihood of COVID-19 vaccine response was negatively associated with both MF diagnosis (57.7% *vs.* 91.7%; *p* = 0.002) and ruxolitinib therapy (66.7% *vs.* 87.5%; *p* = 0.05). A lower response in MF patients was evident either in those not taking ruxolitinib (MF: 6/9 = 66.7% responders *vs.* 22/23 = 95.7%; *p* = 0.03) or in those treated with ruxolitinib (MF: 9/17 = 52.9% responders *vs.* 11/13 = 84.6%; *p* = 0.07). This result for the diagnosis of MF was also confirmed in the multivariable analysis (RR 0.65, 95% CI: 0.44–0.97; *p* = 0.03). In contrast, we found little difference between ruxolitinib-treated patients in both the MF subgroup (*p* = 0.50) and in other MPN patients (*p* = 0.25). The adjusted RR for ruxolitinib was 0.85 (95% CI: 0.58–1.24; *p* = 0.40). Thus, the apparent lower response rate in ruxolitinib-treated patients in the univariate analysis can be explained by “confounding by indication”, since MF represents an indication for treatment with this drug: indeed, 17/26 (65.4%) of MF patients were treated with ruxolitinib *vs.* 13/36 (36.1%) of other MPN cases. Interestingly, neither time from MPN diagnosis to vaccination, nor time from start of ongoing therapy to vaccination seem to impact on vaccine response.

**Table 2:** Predictors of response to vaccine in *BCR-ABL1*-negative MPN patients.

Variable	No. of patients	No. (%) of responders	Anti-spike (U/mL) median
All	62	48 (77.4)	743
*Vaccine type*			
Moderna	40	29 (72.5)	761
Pfizer-BioNTech	22	19 (86.4)	726
*p*-Value		0.21	0.32
*Gender*			
Men	35	25 (71.4)	726
Women	27	23 (85.2)	796
*p*-Value		0.19	0.35
*Age at vaccination*			
<70 years	29	24 (82.8)	1007
≥70 years	33	24 (72.7)	208
*p*-Value		0.34	**0.03**
*Body mass index (kg/m*^*2*^*)*			
<25	35	27 (77.1)	726
≥25	27	21 (77.8)	761
*p*-value		0.95	0.90
*Diagnosis*			
Other MPN	36	33 (91.7)	885
Myelofibrosis	26	15 (57.7)	187
*p*-Value		**0.002**	**0.0001**
*Neutrophils count*			
≤5 × 10^9^/L	28	23 (82.1)	278
>5 × 10^9^/L	34	25 (73.5)	885
*p*-Value		0.42	0.55
*Lymphocytes count*			
≤1.5 × 10^9^/L	33	27 (81.8)	278
>1.5 × 10^9^/L	29	21 (72.4)	1688
*p*-Value		0.38	0.37
*Time from MPN diagnosis to vaccination*			
<10 years	33	25 (75.8)	726
≥10 years	29	23 (79.3)	761
*p*-Value		0.74	0.57
*Time from start of ongoing therapy to vaccination*			
<3 years	25	18 (72.0)	170
≥3 years	32	26 (81.3)	1574
*p*-Value		0.40	**0.013**
*Treatment*			
Other	32	28 (87.5)	1958
Ruxolitinib	30	20 (66.7)	148
*p*-Value		**0.05**	**<0.0001**
*Lines of therapy*			
1	24	20 (83.3)	782
>1	33	24 (72.7)	269
*p*-Value		0.34	0.13

^*^From chi-squared (for percentage of responders) or Mann–Whitney (for anti-Spike titer) test.

Bold values indicates statistically significant *p* values.

In univariate analyses, older age, MF diagnosis, ruxolitinib therapy, and short time (<3 years) from start of ongoing therapy to vaccination were all associated with lower anti-Spike levels (Table 2, *fourth column*). However, in the multiple regression model only MF diagnosis (*p* = 0.002) and ruxolitinib (*p* = 0.005) were confirmed.

Regarding the safety profile of SARS-CoV-2 mRNA vaccines in this specific subgroup of patients, no significant adverse event was reported, except for transient fever or pain at the injection site over the next few days in most cases. No patient suffered from thrombotic or bleeding complications after vaccination.

In conclusion, the rate of seroconversion to mRNA SARS-CoV-2 vaccines in MPN patients (77.4%) is lower as compared to adult healthy people (e.g., >99% among workers of our Hospital) [15], with MF patients showing the worst response (<60%). In addition, among responders, median anti-Spike titers were adversely affected by treatment with ruxolitinib. Even though the exact mechanism for this impaired response is not yet known, it might be the result of both disease- and treatment-mediated immune dysfunction. Although clear-cut relationships between specific anti-Spike titers and protection against the virus has not been unequivocally established, MPN patients, in particular those with MF either receiving ruxolitinib or not, should be urged to maintain high levels of protective measures against COVID-19 also after being vaccinated.
